# Caregiver burden and caregiver appraisal of psychiatric symptoms are not modulated by subthalamic deep brain stimulation for Parkinson’s disease

**DOI:** 10.1038/s41531-018-0048-2

**Published:** 2018-04-17

**Authors:** Philip E. Mosley, Michael Breakspear, Terry Coyne, Peter Silburn, David Smith

**Affiliations:** 1 Systems Neuroscience Group, QIMR Berghofer Medical Research Institute, Herston, QLD Australia; 2 Neurosciences Queensland, St Andrew’s War Memorial Hospital, Spring Hill, QLD Australia; 3 Queensland Brain Institute, University of Queensland, St Lucia, Brisbane, QLD Australia; 4 Faculty of Medicine, University of Queensland, Herston, QLD Australia; 5 Brizbrain and Spine, The Wesley Hospital, Auchenflower, QLD Australia

## Abstract

Subthalamic deep brain stimulation is an advanced therapy that typically improves quality of life for persons with Parkinson’s disease (PD). However, the effect on caregiver burden is unclear. We recruited 64 persons with PD and their caregivers from a movement disorders clinic during the assessment of eligibility for subthalamic DBS. We used clinician-, patient- and caregiver-rated instruments to follow the patient–caregiver dyad from pre- to postoperative status, sampling repeatedly in the postoperative period to ascertain fluctuations in phenotypic variables. We employed multivariate models to identify key drivers of burden. We clustered caregiver-rated variables into ‘high’ and ‘low’ symptom groups and examined whether postoperative cluster assignment could be predicted from baseline values. Psychiatric symptoms in the postoperative period made a substantial contribution to longitudinal caregiver burden. The development of stimulation-dependent mood changes was also associated with increased burden. However, caregiver burden and caregiver-rated psychiatric symptom clusters were temporally stable and thus predicted only by their baseline values. We confirmed this finding using frequentist and Bayesian statistics, concluding that in our sample, subthalamic DBS for PD did not significantly influence caregiver burden or caregiver-rated psychiatric symptoms. Specifically, patient–caregiver dyads with high burden and high levels of psychiatric symptoms at baseline were likely to maintain this profile during follow-up. These findings support the importance of assessing caregiver burden prior to functional neurosurgery. Furthermore, they suggest that interventions addressing caregiver burden in this population should target those with greater symptomatology at baseline and may usefully prioritise psychiatric symptoms reported by the caregiver.

## Deep brain stimulation: negligible effect on caregiver burden

Using subthalamic deep brain stimulation (DBS) to treat patients with advanced Parkinson’s disease (PD) does not alleviate caregiver burden. Continuous electrical stimulation of deep brain nuclei has been shown to improve motor symptoms, reduce the need for dopaminergic medication and generally enhance patients’ quality of life. However, DBS can also produce psychiatric side effects, including depression and impulsivity potentially increasing the strain on caregivers. An Australian study led by Philip E. Mosley at Neurosciences Queensland followed the pre- and postoperative status of 64 patients and their caregivers for 26 weeks. They found that DBS did not influence caregiver burden nor caregiver-rated psychiatric symptoms. This study highlights the importance of assessing the well-being of caregivers prior to the patients’ surgery and the need to manage their expectations about the benefits of DBS treatment.

## Introduction

Caregivers make a substantial contribution to the support of people with Parkinson’s disease (PD). In 2014, Australian PD caregivers provided 19 million hours of care, equivalent to $AUD 78.2 million ($USD 59.5 million).^1^ The PD caregiver may need to coordinate multidisciplinary treatment, advocate for additional services, administer medication, assist with personal care, prevent falls and provide emotional support. However, caregivers are at risk of burden, defined as ‘the extent to which caregivers perceive that caregiving has had an adverse effect on their emotional, social, financial, physical and spiritual functioning.’^2^ Burden is associated with adverse psychiatric outcomes amongst caregivers,^3,4^ and may reduce the effectiveness and tolerability of caregiving, resulting in earlier use of state-sponsored services or premature institutionalisation. PD is a complex disorder manifesting motor and non-motor symptoms, both of which may amplify caregiver burden. Psychiatric symptoms, including depression, anxiety, apathy, psychosis, cognitive impairment and impulse-control disorders (ICDs) have consistently been associated with higher levels of burden.^5^ The cumulative prevalence of psychiatric and cognitive comorbidity in PD is estimated to be >50%,^6^ with contributions from neurodegeneration, adverse effects of treatment and psychological reactions to progressive disability.

Deep brain stimulation (DBS) is an advanced therapy for PD that involves neurosurgery to position electrodes in deep brain nuclei. These produce continuous electrical stimulation to modulate disordered basal ganglia activity. Individuals with motor complications of drug therapy that receive DBS may have a better outcome than those maintained on medication alone, expressed as an improvement in motor symptoms, a reduced requirement for dopaminergic medication and a better self-rated quality of life.^7,8^ However, DBS is not a treatment for psychiatric symptoms in PD, which may continue to progress postoperatively. Furthermore, new psychiatric problems may also emerge, related to the titration of stimulation, the withdrawal of dopaminergic medication and to the inevitable psychosocial adaptation that follows relief of disability in a patient–caregiver dyad.^9^ (Note, although we prefer to use the term ‘person with PD’, we occasionally employ the term ‘patient’ when this role is contrasted with that of ‘caregiver’).

The subthalamic nucleus (STN) is the most common surgical target for DBS in Australasia. However, the anatomy of this nucleus confers vulnerability to stimulation-dependent cognitive and affective disinhibition.^10–12^ Accordingly, some persons with PD become more impulsive and less empathic after DBS, acting recklessly without foresight or concern for others, potentially increasing caregiver burden.^13^ The incidence of this syndrome has been estimated at between 1 and 15%.^14,15^

Early reports noted relational conflicts subsequent to STN-DBS, linked to perceived behavioural changes in the person with PD, despite a good motor outcome and in the absence of significant relational difficulties prior to DBS.^16,17^ Prior research has suggested that as many as 50% of caregivers rate their wellbeing as negative following STN-DBS, despite positive patient-rated outcomes. Psychiatric symptoms are significant covariates of negative caregiver ratings.^18^ Importantly, patient and caregiver ratings of postoperative affective changes are frequently discrepant.^19^ Despite its clinical importance, the main factors influencing post-DBS caregiver burden, indeed whether burden increases or decreases after DBS, remain unclear.

The objective of this study was to examine the trajectory and determinants of caregiver burden in a consecutive sample of persons with PD referred for STN-DBS at one DBS centre in Australia. We employed a prospective, longitudinal design with repeated-measures sampling to capture fluctuations in motor and psychiatric symptoms as the patient–caregiver dyad progressed from pre- to postoperative status. A longitudinal investigation enables more accurate inference on the direction of causality and the temporal evolution of predictor variables. Furthermore, in this cohort, postoperative symptoms may be temporally connected to modulation of stimulation and withdrawal of dopaminergic medication, which may not be captured in a simple pre-post design. Additionally, in this investigation we specifically sought the perspective of the caregiver in rating psychiatric outcomes, motivated by clinical experience, which suggests that persons with PD may less aware of emerging affective and cognitive changes. We hypothesised that in addition to postoperative motor symptoms, psychiatric symptoms would be significant determinants of caregiver burden. Of these, we conjectured that higher impulsivity and lower empathy would be the most significant psychiatric factors. Furthermore, we hypothesised that higher levels of burden would be present in caregivers of persons with PD developing stimulation-dependent mood changes requiring intervention from a psychiatrist.

## Results

### Demographic and baseline variables

Sixty-eight eligible patient–caregiver dyads were approached between 2013 and 2017, and 64 subsequently consented to participate in the study. No participants withdrew from the investigation. Across all data points, <5% was missing for any variable across the investigation. The sample of persons with PD was comprised of predominantly male (48 males and 16 females), predominantly middle-aged individuals with most being classified as the ‘akinetic-rigid’ or ‘mixed’ phenotype. Most had moderate motor symptoms at baseline despite being ‘ON’ medication during Unified Parkinson’s Disease Rating Scale (UPDRS) assessment. Persons with PD displayed a large range in measures of impulsivity and empathy, but generally evidenced mild symptoms of depression, anxiety and apathy at baseline. Caregivers were predominantly female (48 females and 16 males) and were all informal family caregivers, residing with the person with PD. Sixty-three caregivers were spouses and one was the adult child of the person with PD. The mean age of caregivers was 58.3 (SD 8.4). Caregivers also displayed a large range in their ratings of burden, relationship quality and psychiatric symptoms (Table 1).

**Table 1:** Characteristics of STN-DBS patients at baseline

	Akinetic-rigid		Tremor		Mixed		Total	
Gender	*N*	% Total	*N*	% Total	*N*	% Total	*N*	%
Male	19	29.7	10	15.6	19	29.7	48	75
Female	5	7.8	2	3.1	9	14.1	16	25
Mean (SD), median (range)								
Age (years)	65.9 (±7.8), 68 (47–76)		58.2 (±9.3), 61 (35–67)		60.7 (±10.1), 62 (40–77)		62.2 (±9.5), 65 (35–77)	
Hoehn and Yahr stage	2.7 (±0.5), 2.5 (2–4)		2.5 (±0.5), 2.5 (2–3)		2.8 (±0.5), 3 (1.5–4)		2.7 (±0.5), 2.5 (1.5–4)	
Years since diagnosis	8.8 (±5.3), 7 (1–23)		8.4 (±6.2), 6 (2–22)		9.4 (±4.9), 8 (3–21)		9.0 (±5.2), 7 (1–23)	
Levodopa equiv. daily dose	1136.8 (±632.2), 1013 (458–3450)		892.6 (±376.5), 903 (75–1400)		1079.6 (±542.3), 1124 (0–2200)		1066 (±551.8), 1000 (0–3450)	
Patient-rated BIS	60.2 (±9.9), 60 (43–87)		59.1 (±7.5), 60 (44–74)		61.0 (±7.7), 60 (45–76)		60.3 (±8.4), 60 (43–87)	
Caregiver-rated BIS	56.0 (±13.2), 52 (40–90)		62.4 (±10.1), 64 (42–77)		60.6 (±10.5), 61 (44–90)		59.2 (±11.7), 59 (40–90)	
Patient-rated EQ	42.5 (±13.3), 42 (17–68)		43.0 (±13.5), 41 (23–67)		40.6 (±10.9), 41 (16–62)		41.8 (±12.2), 41 (16–68)	
Caregiver-rated EQ	41.2 (±13.1), 42 (13–61)		35.8 (±18.0) 29 (11–64)		37.1 (±12.6), 36 (13–57)		38.4 (±13.9), 39 (11–64)	
QUIP-RS total	18.1 (±15.0), 16 (0–55)		18.0 (±9.5), 18 (0–35)		24.5 (±16.0), 21 (0–63)		20.9 (±14.8), 19 (0–63)	
Beck Depression Inventory	11.4 (±5.3) 12 (1–21)		9.3 (±5.7), 9 (1–20)		12.2 (±4.5), 13 (4–22)		11.3 (±5.1), 12 (1–22)	
Geriatric Anxiety Inventory	3.9 (±3.7), 4 (0–17)		4.6 (±4.1), 4 (0–12)		5.5 (±4.6), 5 (0–15)		4.8 (±4.2), 5 (0–17)	
Apathy scale	11.4 (±5.4), 12 (1–22)		10.6 (±6.4), 10 (1–23)		11.9 (±5.4), 11 (3–26)		11.5 (±5.5), 11 (1–26)	
Mini-Mental State Examination	28.1 (±1.6), 28 (25–30)		28.6 (±1.4), 29 (25–30)		28.6 (±1.4), 29 (25–30)		28.4 (±1.5), 29 (25–30)	
Montreal Cognitive Assessment	26.3 (±2.7), 27 (21–30)		26.1 (2.6), 28 (21–29)		25.8 (±3.2), 27 (16–29)		26.0 (±2.9), 27 (16–30)	
Hayling AB Error score	11.5 (±11.9), 7 (0–38)		8.2 (±10.6), 2 (0–33)		9.8 (±11.8), 4 (0–45)		10.1 (±11.5), 5 (0–45)	
Excluded Letter Fluency rule violations	9.2 (±6.1), 7 (3–24)		8.6 (±6.5), 8 (0–21)		8.3 (±4.3), 8 (2–17)		8.7 (±5.4), 8 (0–24)	
Delay discount *k*	0.016 (±0.022), 0.0079 (0.00016–0.1)		0.019 (±0.019), 0.016 (0.00016–0.064)		0.041 (±0.076), 0.0098 (0.00016–0.25)		0.027 (±0.054), 0.0098 (0.00016–0.25)	
UPDRS Part III Motor Examination	33.3 (±13.6), 31 (13–60)		33.1 (±15.5), 37 (10–51)		42.6 (±18.7), 39 (17–91)		37.3 (±16.8), 36 (10–91)	
Zarit Burden Inventory	19.0 (±13.6), 18 (1–54)		19.4 (±15.4), 19 (2–47)		24.4 (±13.0), 24 (0–49)		21.4 (±13.7), 21 (0–54)	
Relationship Quality Inventory	39.8 (±5.7), 42 (27–45)		36.5 (±8.8), 39 (16–45)		36.7 (±7.0), 37 (22–45)		37.8 (±7.0), 40 (16–45)	

See Methods for full description of instruments

*BIS* Barratt Impulsiveness Scale, *EQ* Empathy Quotient, *QUIP-RS* Questionnaire for Impulsive-Compulsive Disorders in Parkinson’s Disease Rating Scale, *UPDRS* Unified Parkinson’s Disease Rating Scale

### Multivariate modelling of caregiver burden

We were interested in characterising those factors most predictive of caregiver burden (as measured by the Zarit Burden Interview (ZBI)) in our longitudinal sample. We performed an exhaustive multivariate analysis on all possible combinations of candidate covariates in a longitudinal mixed-effects model to identify those with prognostic value for longitudinal burden, with scores on the ZBI as the dependent variable. Our results demonstrated a substantial contribution of psychiatric symptoms to caregiver burden. We found statistically significant positive associations with ZBI for depressive symptoms, as rated by the Beck Depression Inventory (BDI); attentional impulsiveness (caregiver-rated), as rated by the attentional subscale of the Barratt Impulsiveness Scale (BIS); impaired set-shifting and prepotent inhibition, as measured by Hayling Category A Errors (a marker of significant disinhibition); hypersexuality, as measured by the sex subscale of the Questionnaire for Impulsive-Compulsive disorders in PD Rating Scale (QUIP-RS); dopaminergic medication dose, as quantified by the levodopa equivalent daily dose (LEDD); and PD motor symptoms, as rated by the UPDRS Part III Motor Examination (Table 2). Caregiver ratings of relationship quality, as measured by the Relationship Quality Index (RQI), had a negative correlation with ZBI, with higher levels of burden associated with lower relationship quality. Caregiver ratings of empathy, as measured by the Empathy Quotient (EQ), also demonstrated a negative correlation, indicating that as empathy decreased, burden increased. In a second approach, we also applied a variable selection and regularisation algorithm (the Least Absolute Shrinkage and Selection Operator: LASSO) to identify the combination of prognostic covariates that best predicted longitudinal ZBI without overfitting. Setting of a conservative regularisation term penalised model complexity. The LASSO showed agreement with the exhaustive multivariate analysis, although it included clinical subtype (akinetic-rigid with higher ZBI) and set-shifting as measured by the Excluded Letter Fluency (ELF) rule violations (also a marker of disinhibition), while dropping LEDD.

**Table 2:** Results of multivariate modelling for caregiver burden

Variable	Direction of association	Chi square value	Significance
RQI total	−	60.7	6.5 × 10^−15^***
Caregiver-rated BIS attentional	+	14.7	0.00013***
BDI total	+	14.2	0.00016***
QUIP-RS hypersexuality	+	9.0	0.0027**
Caregiver-rated EQ	–	7.7	0.0057**
Log_0_ LEDD	+	6.0	0.026*
UPDRS Part III total	+	4.9	0.027*
Hayling Category A errors	+	4.5	0.035*

See Methods for full description of instruments

*BDI* Beck Depression Inventory, *BIS* Barratt Impulsiveness Scale, *EQ* Empathy Quotient, *LEDD* levodopa equivalent daily dose (natural logarithm employed due to nonparametric distribution), *QUIP-RS* Questionnaire for Impulsive-Compulsive Disorders in Parkinson’s Disease Rating Scale, *RQI* Relationship Quality Index, *UPDRS* Unified Parkinson’s Disease Rating Scale

*p < 0.05; **p < 0.01; ***p < 0.001

We noted the likelihood of complex interactions between predictors and extended the optimal multivariate models to test for interaction effects up to second order. Our genetic algorithm reported on the top 20 candidate models and then we performed backwards elimination to reduce the number of parameters and interaction terms. Only 1 statistically significant interaction term was identified, between the caregiver-rated BIS attentional subscale and Hayling Category A errors (*p* < 0.0088).

### Burden associated with stimulation-related psychiatric symptoms

**Fig. 1:**
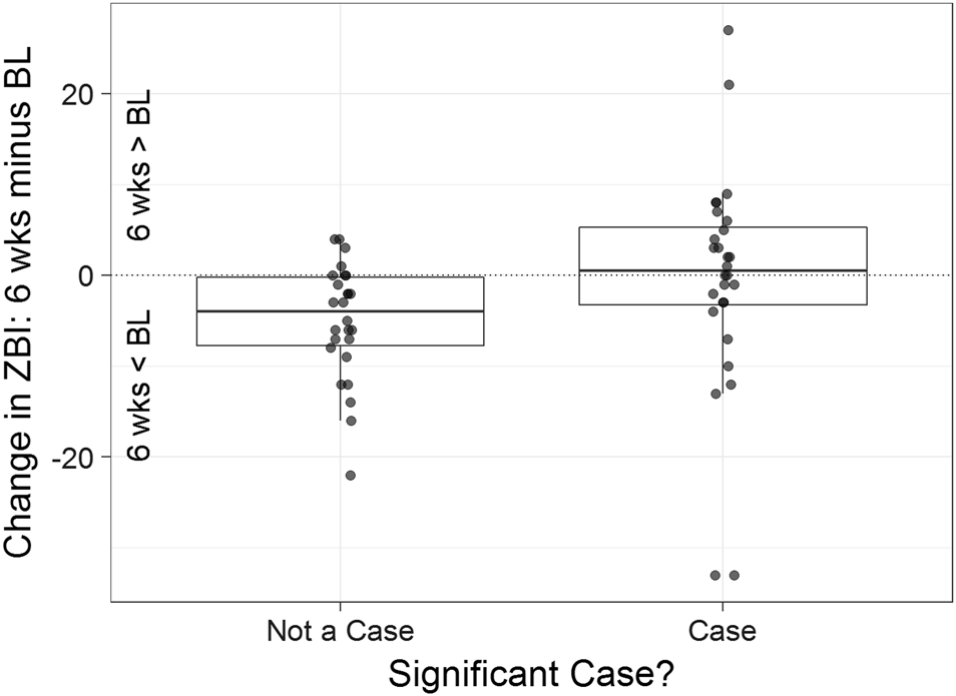
Developing psychiatric symptoms attributable to neurostimulation in the first 6 weeks post DBS is associated with significantly greater caregiver burden. BL baseline, ZBI Zarit Burden Inventory. Thick bar = mean change in ZBI at 6 weeks compared to baseline, box = standard deviation

We wished to test whether caregiver burden was increased by changes in mood and behaviour that can arise during the early postoperative phase of subthalamic DBS titration. We tested for differences in caregiver-rated burden and psychiatric symptoms between those persons with PD who developed new psychiatric symptoms due to subthalamic stimulation (‘cases’) and those who did not (‘non-cases’). Classification of ‘caseness’ was undertaken by a psychiatrist and neurologist (see Methods for details). We compared two epochs: from baseline versus 2 weeks and baseline versus 6 weeks. We employed ratings of caregiver burden (ZBI), caregiver-rated empathy (EQ) and caregiver-rated impulsiveness (BIS). We extended this analysis to a linear model that included clinical subtype as an interaction factor with cases. Of these analyses, we found a statistically significant difference in burden between cases and non-cases at 6 weeks post-DBS (*p* = 0.0005, Fig. 1). There was a similar trend towards a difference in caregiver-rated EQ between cases and non-cases at 6 weeks that did not reach significance (*p* = 0.0550). We found no significant differences between cases and non-cases from baseline to the 2- and 6-week follow-up times with respect to caregiver-rated BIS (*p* = 0.96 and 0.43, respectively), nor were there any interactions between cases and tremor versus akinetic-rigid or mixed subtype (*p* = 0.87 and 0.75, respectively).

### Longitudinal trajectories of caregiver-rated variables

We desired to investigate whether caregiver burden and caregiver ratings of psychiatric symptoms were longitudinally affected by their partner undertaking subthalamic DBS. We first employed a repeated-measures analysis of variance (ANOVA), which demonstrated an expected and significant longitudinal reduction in PD motor symptoms and the requirement for dopaminergic medication. There was also a significant reduction in depressive symptoms in the person with PD and a small, but significant, decrease in caregiver-rated relationship quality. There were no other significant longitudinal changes in patient-, caregiver- or clinician-rated variables at a group level. There were also no significant group changes in relevant subscales (such BIS second-order factors, or QUIP-RS subscales; Table 3).

**Table 3:** Repeated-measures analysis of variance

Instrument	*F* statistic	Significance
Apathy scale	0.838	0.502
BIS	0.757	0.554
BDI	5.865 (decrease)	0.000146***
EQ	0.273	0.895
GAI	1.89	0.112
QUIP-RS	1.383	0.24
Caregiver-rated BIS	0.768	0.546
Caregiver-rated EQ	1.291	0.273
RQI	2.881 (decrease)	0.0229*
ZBI	0.276	0.894
Delay discount *k*	0.443	0.778
ELF rule violations	0.104	0.981
Hayling AB error score	0.81	0.519
Mini-Mental State Examination	1.055	0.379
Montreal Cognitive Assessment	0.454	0.769
UPDRS Part III total	13.5 (decrease)	5.54 × 10^−10^***
LEDD	62.73 (decrease)	2 × 10^−16^***

See Methods for full description of instruments

*BDI* Beck Depression Inventory, *BIS* Barratt Impulsiveness Scale, *ELF* Excluded Letter Fluency, *EQ* Empathy Quotient, *GAI* Geriatric Anxiety Inventory, *LEDD* levodopa equivalent daily dose, *QUIP-RS* Questionnaire for Impulsive-Compulsive Disorders in Parkinson’s Disease Rating Scale, *RQI* Relationship Quality Index, *UPDRS* Unified Parkinson’s Disease Rating Scale, *ZBI* Zarit Burden Interview

***p < 0.001; **p < 0.01; *p < 0.05

However, suspecting considerable heterogeneity in our sample, we further examined individual participant-wise trajectories, focussing on caregivers. We selected the longitudinal trajectories of ZBI, caregiver-rated EQ and caregiver-rated BIS. We clustered individuals based on their longitudinal trajectories, using both a frequentist and a Bayesian approach. The advantage of the Bayesian approach was the estimation of posterior probabilities of cluster assignment for each caregiver rating at each time point, in addition to the likelihood of transition between clusters—i.e., how stable was a caregiver’s rating of burden and psychiatric symptoms as they moved through each postoperative interval?

**Fig. 2:**
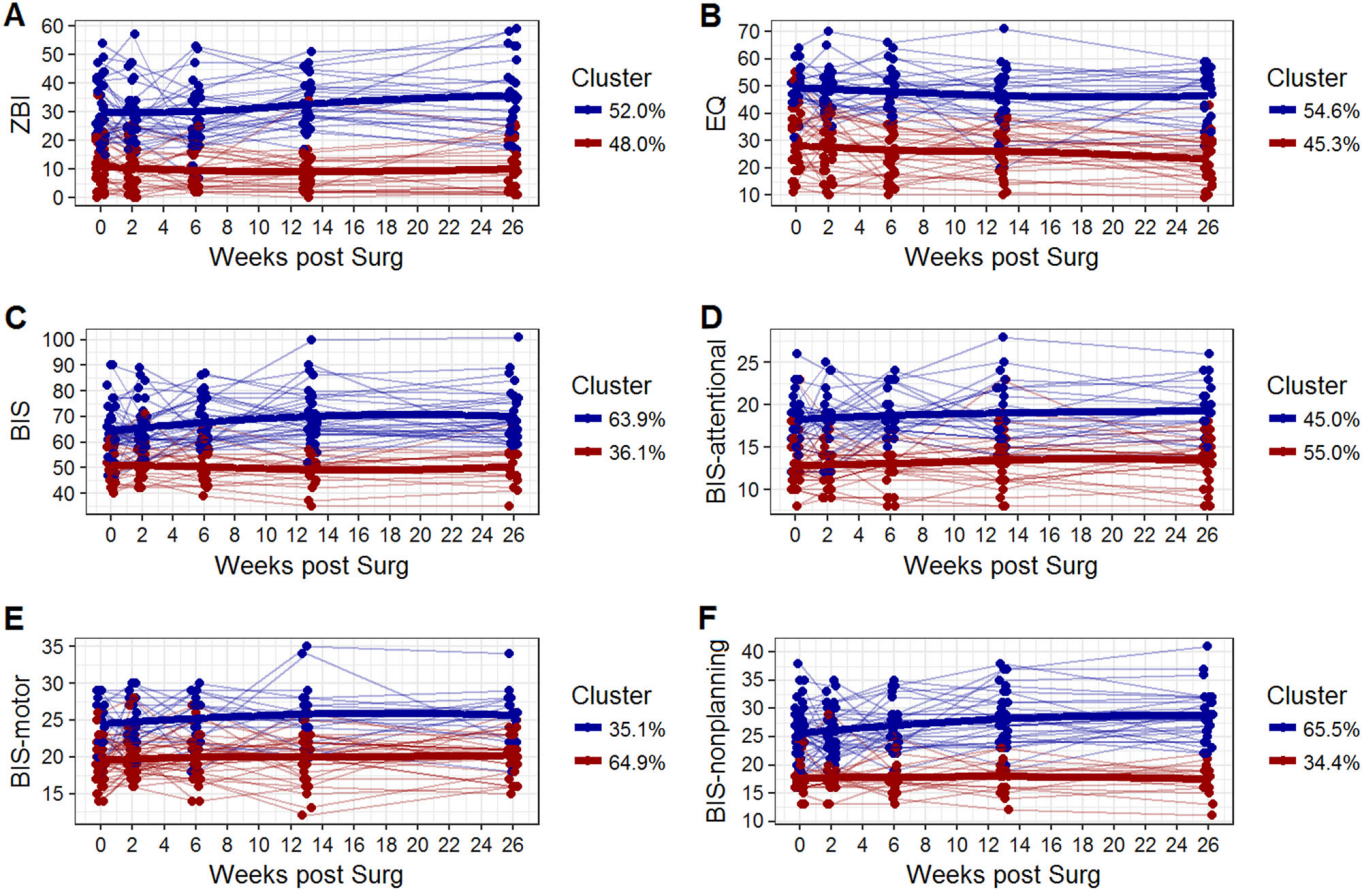
Frequentist clustering of the caregiver-rated variables ZBI, caregiver-EQ and caregiver-BIS (plus subscales). The line segments represent individual trajectories of each caregiver’s rating, and the thick lines are LOESS-smoothed cluster-wise trajectories. The percentages are the proportion of caregivers assigned to each of the two clusters. BIS Barratt Impulsiveness Scale, EQ Empathy Quotient, ZBI Zarit Burden Inventory

We considered the existence of up to five clusters. According to both approaches, the optimal number of clusters was two across all caregiver endpoints. We categorised these as ‘high’ and ‘low’ clusters, noting that the ‘high’ cluster was the ranking relative to the ‘low’ and as such might include normal or impaired functioning on a given instrument. The cluster assignments of the frequentist approach across six caregiver endpoints are shown in Fig. 2. Note that ‘high’ and ‘low’ clusters are well differentiated longitudinally with little egress of one cluster from its group trajectory into the path of another. The frequentist analysis of caregiver burden (ZBI score) identified a ‘high’ cluster with a longitudinal mean of 31.09 (SD 7.04) and a ‘low’ cluster with a longitudinal mean of 10.83 (SD 5.88). The 95% confidence interval between ZBI scores across these clusters was 17.02–23.49 (*t* = 12.54, *p* = 2.2 × 10^−16^). Descriptive statistics for caregiver-rated EQ and BIS are presented in Supplementary Table 1.

**Fig. 3:**
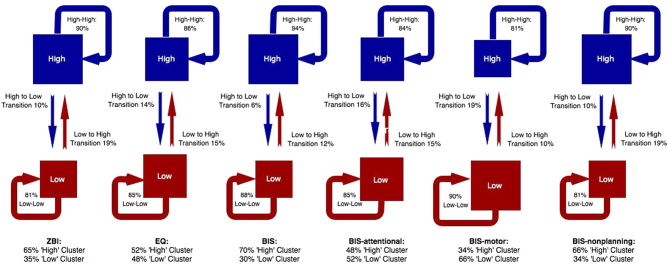
Hidden Markov modelling of cluster assignment and transition probabilities for the caregiver-rated variables ZBI, caregiver-EQ and caregiver-BIS (plus subscales). For each variable, the posterior probability of cluster assignment (‘high’ versus ‘low’) is denoted below the variable heading. Transition probabilities for each cluster are represented by arrows. For example, for ZBI, 65% of the sample are assigned to the ‘high’ cluster. Amongst that cluster, there is only a 10% transition probability from ‘high’ to ‘low’, indicating relative stability of ‘high’ cluster assignment for that variable. BIS Barratt Impulsiveness Scale, EQ Empathy Quotient, ZBI Zarit Burden Inventory

Whereas cluster assignment in the frequentist approach is fixed, the Bayesian framework (through hidden Markov models) permits analysis of longitudinal cluster transition probabilities—that is, the probability of individuals changing clusters over time. Figure 3 shows the posterior probabilities (‘high’ and ‘low’) of cluster assignment per caregiver endpoint, with transition probabilities represented by direct arrows between clusters. The distribution of cluster assignment using the Bayesian framework showed general agreement with the posterior probabilities in the frequentist approach. However, for the caregiver-rated ZBI, EQ, BIS and subscales, transition probabilities between ‘high’ and ‘low’ clusters are small (displayed in Fig. 3). This implies caregivers reporting high levels of burden prior to subthalamic DBS, as well as those caregivers reporting high levels of impulsiveness and low levels of empathy, are unlikely to change this profile subsequent to subthalamic DBS. In other words, the pre-DBS score for a given variable is likely to be similar to the score post-DBS and during follow-up.

We strengthened this finding in a machine-learning model (gradient boosting) by looking for baseline factors that predicted for clusters ‘high’ or ‘low’ across the ZBI, EQ, BIS and subscales using all available baseline factors, both patient- and caregiver-rated. When we excluded the associated baseline numeric endpoints in our logistic models, there were no significant pre-DBS factors that predicted for clusters. When we included the relevant pre-DBS measure, these emerged as predictive for their respective cluster. This implies that a caregiver rating prior to DBS is generally reflective of that endpoint’s longitudinal cluster, regardless of all other factors.

Given the negative correlation between RQI and ZBI score in the multivariate analysis, we were interested in whether relationship quality was an important moderating factor determining longitudinal assignment to a ‘high’ or ‘low’ burden cluster. In a general linear model, there was a strong univariate relationship between RQI and ZBI cluster assignment (*z* = 3.2, *p* = 0.0015). However, when other variables from the exhaustive multivariate modelling were added to the model, the contribution of RQI score became non-significant (*z* = 1.74, *p* = 0.082). Only adding baseline ZBI score to this multivariate model proved to be significant (*z* = −3.017, *p* = 0.0026), consistent with the gradient boosting analysis.

To evaluate the contribution of RQI to a classifier of ZBI cluster, we examined the gradient boosting analysis more closely. When the ZBI baseline score was excluded from the classification model, RQI made a significant contribution to cluster assignment (18.8%). However, the accuracy of this model at correctly classifying cluster assignment was poor (0.58, 95% confidence interval 0.34–0.80). When the ZBI baseline score was included, the contribution of RQI to cluster assignment halved (9.01%) but the accuracy of the model was superior (0.89, 95% confidence interval 0.67–0.99). This suggests that in this cohort, relationship quality (as assessed by the RQI) was not a dominant moderating factor influencing caregiver assignment to ‘high’ or ‘low’ burden clusters. However, in a further analysis, we evaluated the likelihood of cluster assignment based on RQI score. Participants had an approximately equal likelihood of ‘high’ or ‘low’ ZBI cluster assignment with scores of 34 or above (maximum score 45). However, when the RQI score fell to 33 or below, the likelihood of assignment to the ‘high’ ZBI cluster rose to 0.8. This suggests that RQI may become an important moderating factor upon caregiver burden at lower values (see Supplementary Figure 1).

## Discussion

We present a longitudinal analysis of caregiver burden in a cohort of extensively phenotyped individuals with PD undertaking STN-DBS. Our data add to the relatively sparse literature in this domain, with the advantage of employing an iterative, longitudinal approach. This confers greater confidence in the direction of associations, as well as a more sophisticated analysis of the trajectories of caregiver-rated endpoints. At each assessment in the longitudinal investigation, a broad range of motor, neurocognitive and neuropsychological instruments were employed to phenotype persons with PD. Subsequently, a multivariate analysis in a longitudinal mixed-effects model identified a significant association between burden and comorbid psychiatric symptoms. Caregiver burden and caregiver ratings of impulsiveness and empathy were subsequently selected for longitudinal analysis using a frequentist and Bayesian approach to identify whether DBS meaningfully altered their trajectories.

### Determinants of caregiver burden

We used an exhaustive, model-based approach to ascertain the minimum set of predictors of caregiver burden (ascertained with the ZBI). Consistent with our hypothesis, psychiatric symptoms made a substantial contribution, in addition to motor symptoms. Depressive symptoms, caregiver-rated empathy, caregiver-rated impulsivity and hypersexuality were significant predictors of ZBI score, whilst measures of prepotent response inhibition on the Hayling and ELF tests were also significant predictors. Our two methods for longitudinal multivariate modelling furnished similar results, suggesting the consistency of these findings. The onset of post-DBS stimulation-related psychiatric symptoms was also associated with significantly greater burden.

### Stability and collinearity of caregiver cluster assignment

We used both a frequentist and a Bayesian method to provide complimentary analyses of the clustering of longitudinal trajectories of caregiver-rated variables and the stability of cluster assignment. We found that each individual variable was optimally parsed into two clusters, with a relatively low probability of switching between clusters during follow-up. Furthermore, the only factor that predicted longitudinal cluster assignment was the numeric observation of that endpoint at baseline. This suggests that STN-DBS in this sample did not substantially alter the caregiver-rated psychiatric phenotype nor whether caregivers were classified into ‘high’ or ‘low’ burden groups, despite considerable changes to medication and neurostimulation over the course of the investigation. Furthermore, longitudinal cluster assignment could not be predicted from the overall psychiatric profile at baseline without collinearity. The longitudinal means of these clusters were significantly different, suggesting their separation was not due to chance or measurement error, but representative of a cohort of caregivers with truly greater burden. Caregiver burden (as measured by the ZBI) in our ‘high’ burden group was of a similar degree to that reported by caregivers of persons with PD with apathy and ICDs,^20^ as well as by caregivers of individuals with a Clinical Impression of Severity Index for PD score of ‘severe’.^3^ This suggests that the burden in this cluster is clinically significant, consistent with prior reports amongst cohorts of significantly impaired persons with PD. Previous work has suggested a cutoff score of 24 or greater on the ZBI as indicative of caregivers at high risk of clinically significant depressive symptoms, requiring further assessment.^21^

## Conclusions

Despite the effectiveness of STN-DBS in the treatment of motor symptoms of PD and its benefits for patient-rated quality of life, its effect on caregiver burden is less clear. In this cohort, longitudinal levels of caregiver burden were not significantly altered post-DBS. Psychiatric symptoms were significant drivers of post-DBS burden and included depression, impulsivity, compulsivity and impulsive responding on neuropsychological tests of prepotent inhibition. When clinically significant levels of stimulation-dependent psychiatric symptoms emerged during the post-DBS period (as evaluated by a psychiatrist), caregivers of patients with these symptoms endorsed greater burden. This indicates a mechanism through which STN-DBS may transiently increase caregiver burden, until stimulation-dependent symptoms are managed with DBS manipulation. Overall, our findings add to the literature suggesting caregiver- and patient-rated quality of life outcomes subsequent to STN-DBS may be discrepant.

Our study is not the first to find caregiver burden unchanged after subthalamic DBS. Previous reports have utilised measures of caregiver strain or quality of life in a simple pre- and postoperative design, with sample sizes of between 12 and 25.^18,22^ However, our investigation is the first to quantitatively examine longitudinal measures of caregiver burden in an adequately powered sample. This method of repeated sampling allows us to study the stability of burden and caregiver ratings of psychiatric symptoms, despite considerable changes to medication and neurostimulation over the course of the investigation. Additionally, we contribute a multivariate, longitudinal, analysis of those factors significantly associated with caregiver burden in this surgical cohort.

Nonetheless, we acknowledge the moderate size of our sample and the single-centre nature of our investigation. However, our sample size is larger than comparable investigations employing such detailed phenotypic characterisation^16,19,22^ and our calculation suggests our study is adequately powered to meet our primary objective. Additionally, the participants in our investigation are similar in terms of clinical severity, demographics and prior treatment to other large neurosurgical centres. Differences between centres in the postoperative prescribing of dopaminergic medication can influence the evolution of psychiatric symptoms, but we included levodopa equivalent dose as a variable in our multivariate modelling and we provide summary statistics describing dopaminergic prescribing at each postoperative time point to aid comparison with other centres (Supplementary Table 2). Moreover, the striking separation and stability of cluster assignment, as evidenced by our frequentist and Bayesian statistics, suggest that the lack of longitudinal change in these variables is likely to be a true effect, rather than resulting from an underpowered cohort.

Future work could incorporate more detailed characterisation of the caregiver, both in terms of demographic status and caregiver psychiatric morbidity. Depressive and anxiety symptoms in the caregiver are strongly associated with burden^5^ and may strengthen the subsequent modelling of postoperative burden in surgical cohorts such as this. Furthermore, the number of years that caregivers have spent in this role may be an important mediating variable to consider. This will permit a more accurate definition of the profile of caregiver at high risk of burden, complementing the present identification of clinical factors in the person with PD that are significantly associated with burden. Our finding that caregivers with poor relationship quality at baseline were highly likely to be assigned to the ‘high’ burden cluster suggests a potential therapeutic target for intervention prior to functional neurosurgery. We suggest that caregivers reporting elevated burden and poor relationship quality at baseline may benefit from a structured psychotherapeutic intervention, beginning prior to DBS and continuing in the early postoperative period. The threshold for entry into the intervention could be determined by a psychiatrist conducting a formal preoperative psychiatric assessment, as part of an assessment of family functioning, in combination with formal rating scales. Cognitive, behavioural and environmental elements contributing to burden could be addressed in a series of modules, targeting such vulnerabilities as maladaptive coping styles, ineffective patterns of interpersonal communication and inadequate social support, all of which may impair patient–caregiver relational functioning. Comprehensive education regarding the role of DBS in the treatment of PD should also be included, in order to prepare caregivers for potential neuropsychiatric complications and ensure that expectations about the benefit of DBS are realistic. We have developed such a manualized intervention, designed to be delivered by a psychologist or specialist nurse, which we are evaluating in a new cohort of caregivers. A similar intervention has been of benefit in caregivers of persons with PD in a non-surgical cohort.^23,24^ Such psychotherapies necessitate extra resources, but the results of this investigation suggest at-risk caregivers can be stratified at baseline, improving the specificity of the intervention.

In summary, caregivers that endorsed high levels of burden at baseline, as well as caregivers that reported high levels of impulsivity and low levels of empathy in their partners, generally maintained this profile during longitudinal follow-up. The converse was also true. Therefore, STN-DBS neither amplified nor ameliorated caregiver burden, nor did it significantly alter caregiver ratings of psychiatric symptoms. Our statistical methods extend standard pre- versus post-test contrasts and allow inference upon individual trajectories, which strengthens our findings. This suggests that clinicians seeking to reduce caregiver burden in this population should identify and target those with high burden at baseline, as these caregivers are most likely to report ongoing burden during their postoperative journey. Furthermore, given the substantial contribution of psychiatric symptoms to postoperative burden, and the stability of psychiatric symptom clusters (as evaluated by caregivers in this paper), a preoperative psychiatric assessment of all surgical candidates is well placed to identify and manage these key variables.

## Methods

Prior to the commencement of data collection, the full protocol was approved by the Human Research Ethics Committees of the Royal Brisbane & Women’s Hospital, the University of Queensland, the QIMR Berghofer Medical Research Institute and UnitingCare Health. All patients and caregivers gave written consent to participate in the study. Sixty-four persons with PD and their caregivers were consecutively recruited at the Asia-Pacific Centre for Neuromodulation, during the assessment of eligibility for STN-DBS. A movement disorders neurologist confirmed the diagnosis of PD, according to the United Kingdom Queens Square Brain Bank criteria.^25^ The laterality of disease onset and the Hoehn and Yahr stage^26^ at operation was recorded. The PD subtype (tremor-dominant, akinetic-rigid and mixed type) was established based on an analysis of the dominant symptoms elicited during the UPDRS Part III Motor Examination, as described in Spiegel et al.^27^ Exclusion criteria were the absence of an identified caregiver, DBS of deep brain nuclei other than the STN and dementia as defined by a Mini-Mental State Examination Score (MMSE) of <25 or a clinical diagnosis of PD dementia. The latter was defined according to the published Movement Disorder Society criteria.^28^ Caregivers were unpaid spouses or family members, residing with the person with PD. Persons with PD and their caregivers were required to be fluent in English in order to participate. Prior to DBS, all patients and their caregivers were assessed by a psychiatrist with experience in movement disorders, who recorded current and historical psychiatric symptoms.

Persons with PD underwent bilateral implantation of Medtronic 3389 or Boston Vercise electrodes in a single-stage procedure. The STN was identified as a midbrain structure on Fluid Attenuation Inversion Recovery imaging and electrodes were targeted to this nucleus using a Leksell stereotactic apparatus. Intraoperative microelectrode recordings were employed to establish localisation within the STN and anaesthesia was down-titrated to perform test stimulation. A computed tomograph scan confirmed satisfactory postoperative lead placement. Stimulation was commenced immediately at low intensity and was titrated over the following week as an inpatient until motor symptoms were satisfactorily controlled. Post discharge, persons with PD returned to the movement disorders clinic for further neurological and psychiatric evaluation, with further DBS manipulation according to a set schedule of visits. The predominant criterion for DBS manipulation at each visit was manifest motor symptoms of PD. However, if the patient, caregiver or clinician detected any new psychiatric symptoms (such as mood elevation, disinhibition or irritability) then a psychiatric review was initiated. Manipulation of the DBS device subsequently occurred if the symptoms were determined to be stimulation-related.^9^

### Measures

Assessments took place prior to DBS and subsequently at 2, 6, 13 and 26 weeks postoperatively, using the same battery of measures. Persons with PD were ‘ON’ medication and stimulation for all assessments. Participants were administered the following neuropsychological instruments, designed to capture the broad range of psychiatric symptoms observable in this cohort: the Barratt Impulsiveness Scale (BIS 11) and second-order factors attentional, motor and non-planning;^29^ the Beck Depression Inventory (BDI II);^30^ the Empathy Quotient (EQ);^31^ the Geriatric Anxiety Inventory;^32^ the Questionnaire for Impulsive-Compulsive Disorders in PD Rating Scale (QUIP-RS);^33^ and the Apathy scale.^34^

In addition to this psychiatric profile, impulsivity was assessed by administration of the following neuropsychological tests: the delay discounting task;^35^ the Excluded Letter Fluency (ELF) task;^36^ and the Hayling test.^37^ The delay discounting task assesses delay aversion, the tendency to prefer sooner, smaller rewards over those that are larger but temporally more distant. It was designed to assess impulsivity in individuals with substance use disorders;^35^ behaviours that share face validity with the ICDs observed in a subset of persons with PD. Both the Hayling and the ELF assess prepotent response inhibition, which is known to be modulated by STN-DBS.^38,39^

Dementia was an exclusion criterion upon entry to the investigation, but it is conceivable that cognitive impairment could emerge over the course of the study as a consequence of disease progression, with associated burden.^40^ In order to assess basic cognitive status, the following additional cognitive tests were also administered: Mini Mental State Examination (MMSE);^41^ and the Montreal Cognitive Assessment (MOCA).^42^

Caregivers completed the Zarit Burden Interview (ZBI)^43^ and the Relationship Quality Index (RQI)^44^ at each visit. In addition, a modified version of the BIS and EQ (caregiver-rated BIS and caregiver-rated EQ) assessed these behavioural domains from the perspective of the caregiver, given that impaired insight may affect patients’ own ratings. This approach has been employed in studies involving PD participants.^45^

At each visit motor symptoms were assessed using the UPDRS Part III Motor Examination. Dopaminergic medication was recorded and converted to a levodopa-equivalent daily dose (LEDD) value.^46^ DBS parameters were also recorded (active contacts, amplitude, pulsewidth and frequency).

Persons with PD remained under the care of a psychiatrist throughout the study. Those who developed clinically significant mood or behaviour changes attributable to STN stimulation were defined as ‘cases’. This was operationalised as follows: the person with PD, their caregiver or a clinician raised concern about new behaviours that were ‘out of character’. The person with PD undertook a semi-structured psychiatric interview with attention to mood elevation, disinhibition, loss of empathy and irritability. If emergent symptoms were detected, the neurologist reduced the amplitude of stimulation or changed the position of the active electrode contact. If symptoms immediately remitted or substantially reduced upon repeated psychiatric assessment, then the symptoms were judged to be stimulation-related. All ‘cases’ subsequently underwent further DBS reprogramming to address these symptoms and remained under close follow-up with the psychiatrist and neurologist until their psychiatric symptoms had entirely remitted (Table 4).

**Table 4:** Full battery of tests and their acronyms

Instrument	Rater	Acronym
Apathy scale	Patient	Apathy scale
Barratt Impulsiveness Scale	Patient	BIS
Beck Depression Inventory II	Patient	BDI
Empathy Quotient	Patient	EQ
Geriatric Anxiety Inventory	Patient	GAI
Questionnaire for Impulsive-Compulsive Disorders in PD Rating Scale	Patient	QUIP-RS
Barratt Impulsive Scale—Caregiver Version	Caregiver	Caregiver-rated BIS
Empathy Quotient—Caregiver Version	Caregiver	Caregiver-rated EQ
Relationship Quality Inventory	Caregiver	RQI
Zarit Burden Interview	Caregiver	ZBI
Delay Discounting Test	Clinician	Delay discounting
Excluded Letter Fluency Test	Clinician	ELF
Hayling Test	Clinician	Hayling
Mini-Mental State Examination	Clinician	MMSE
Montreal Cognitive Assessment	Clinician	MOCA
Unified Parkinson’s Disease Rating Scale Part III Motor Examination	Clinician	UPDRS

### Statistical analysis

#### Sample size calculation

For the assessment of caregiver burden using the ZBI, a difference of 14 points on the ZBI was deemed clinically significant, as this was the difference in means reported by caregivers of PD patients with ICDs (mean 30, SD 14) and non-impulsive PD patients (mean 16, SD 11).^20^ The phenotype of the psychiatric and behavioural change that may arise subsequent to subthalamic DBS, in a proportion of persons with PD, overlaps with the construct of ICDs; so this study was selected as a reasonable proxy for the sample size calculation, noting that no equivalent quantitative data on caregiver burden after subthalamic DBS was available.

A Hedges *g* was calculated, assuming a distribution of cases (as defined above) to non-cases of 20:80, to give an effect size of 1.2. Sample size was calculated using G*Power (version 3.1.9.3),^47^ assuming an independent two-sample *t*-test, with an alpha of 0.05 and a power of 0.90, yielding a total of 56 required participants.

#### Longitudinal determinants of burden

The study design included longitudinal assessments for subjects and their respective caregivers. The objective was to elucidate the trajectory of the caregiver-reported data and identify associated factors with prognostic value. To achieve this, the longitudinal data analysis required estimation of the correlations between assessments for each patient–caregiver dyad. For each caregiver endpoint, we fitted a random intercept-slope mixed-effects longitudinal model and used variable selection techniques to assess associations with prognostic factors.

Variable selection techniques identify the minimum set of prognostic factors and avoid overfitting in the multivariate longitudinal models. Two distinct variable selection methods were employed. The first was the LASSO.^48^ The tuning parameter (*λ*) was defined by the conservative and parsimonious one-standard-deviation rule.^49^ The second method was an exhaustive approach, which scanned a complete set of all combinations of candidate covariates; the model space was then scored and sorted by the Bayesian information criteria. Model space was optimised using a genetic algorithm to identify a reduced model space that included sets of covariates with large effect sizes. Subsequently, after eliminating those models that included covariates with poor associations, an exhaustive search was performed on the reduced model space to consider all combinations of candidate covariates.

Covariates in longitudinal mixed models assume approximately normal distribution for best performance. Several covariates showed positive skewness when summary statistics and histograms were inspected. These covariates included Hayling Errors, ELF repetitions and rule violations, delay discount parameter *k* and LEDD. A natural logarithmic transformation of log (1 + covariate) was applied to these skewed covariates before they were included as candidate prognostic factors in the multivariate longitudinal mixed models. For convenience, we refer to these log-transformed covariates as log_0_ covariate (Table 5).

**Table 5:** Candidate variables included in multivariate modelling of longitudinal ZBI

Patient-reported	Caregiver-reported	Clinician acquired
Apathy total	Caregiver-rated BIS total	Age
BIS total	Caregiver-rated BIS attentional subscale total	Gender
BIS attentional subscale total	Caregiver-rated BIS motor subscale total	Hoehn and Yahr stage
BIS motor subscale total	Caregiver-rated BIS non-planning subscale total	Log_0_ LEDD
BIS non-planning subscale total	Caregiver-rated EQ total	Tremor-akinesia subtype
BDI total	RQI total	Log_0_ delay discount *k*
EQ total	ZBI total	Log_0_ ELF repetitions
GAI total		Log_0_ ELF rule violations
QUIP-RS—buying		Log_0_ Hayling Category A errors
QUIP-RS—eating		Log_0_ Hayling Category B errors
QUIP-RS—gambling		Log_0_ Hayling AB error score
QUIP-RS—hobbyism		MMSE total
QUIP-RS—medication use		MOCA total
QUIP-RS—sex		UPDRS total
QUIP-RS—ICD total		
QUIP-RS—QUIP total		

#### Longitudinal trajectories of burden and caregiver-rated psychiatric symptoms

The objective in this analysis was to model the longitudinal evolution of burden and psychiatric symptoms. A repeated-measures ANOVA was performed for all longitudinal variables but this approach did not account for the observed inter-subject heterogeneity in our sample and could not describe the longitudinal course of our subjects over time. Therefore, we employed more complex modelling of individual trajectories, focussing on caregiver-rated variables for this analysis. Again, using two distinct statistical methods, variables were parsed into separate clusters based on their observed longitudinal trajectories.

A frequentist longitudinal clustering method estimated the number of clusters and each subject’s cluster assignment using *k*-means criteria.^50^ In this implementation, relative indices of the Calinski-Harabasz metric were compared, applying a minimum of three redraws per fixed cluster number using vote counting of the highest Calinski-Harabasz metric to determine the optimal cluster number.^51^

In a separate analysis, clusters were estimated using a Bayesian method, incorporating hidden Markov models and transition probabilities.^52^ Similar to the frequentist clustering approach, the number of clusters was first estimated using a series of steps to optimise an information criterion. Subsequently, the probabilities of switching between or remaining in each cluster were then estimated. Non-informative priors were assigned to the transition probabilities and posterior probabilities for cluster assignments were then estimated. This extends the frequentist longitudinal clustering analysis by estimating the posterior probability that a given caregiver or patient will be found in a given cluster at a given time point and also provides a measure of the relative stability of cluster assignment.

We extended this analysis to find baseline factors that predicted for these longitudinal clusters amongst the caregiver-rated endpoints using generalised boosted regression modelling. In other words, we considered predicting for clusters using all available baseline factors, both patient- and caregiver-rated. We used a training and testing validation approach to find appropriate factors.

In the first set of analyses, we excluded the baseline numeric observations that corresponded to the endpoint. For example, we excluded the pre-DBS caregiver-rated BIS numeric observations when we tried to predict for caregiver-rated BIS clusters. This is strong control against collinearity. In the second set of analyses, we added in these observations as predictors.

### Data availability

All analyses took place in the R software environment,^53^ using the following packages: *lme4* and *nlme for* longitudinal mixed models,^54,55^ *glmnet* for LASSO,^48^ *glmulti* for genetic algorithm model selection,^56,57^ *depmixS4* for hidden Markov models^58^, *kml* for longitudinal clustering^59^ and *gbm* for boosted regression modelling.^60^ Across the complete data set, <5% of data were missing. Missing data were inferred using the pooled results of 50 iterations of imputation by classification and regression trees, employing *mice* for longitudinal data imputation using Gibbs sampling.^61^ A de-identified data set used in the analysis can be requested from the lead author, subject to institutional review board approval.

### Ethical approval

Prior to the commencement of data collection, the full protocol was approved by the Human Research Ethics Committees of the Royal Brisbane & Women’s Hospital, the University of Queensland, the QIMR Berghofer Medical Research Institute and UnitingCare Health. All patients and caregivers gave written consent to participate in the study.

## Electronic supplementary material

Supplementary Material

Supplementary Figure 1
