# Supplementary material for: Caregiver burden and caregiver appraisal of psychiatric symptoms are not modulated by subthalamic deep brain stimulation for Parkinson’s disease
Source: NPJ Parkinsons Dis. 2018 Apr 17;4:12. doi: 10.1038/s41531-018-0048-2 (PMC5904120; doi:10.1038/s41531-018-0048-2)
Supplement: Supplementary file 1 — Supplementary Material [file 41531_2018_48_MOESM1_ESM.pdf]

Supplementary Table 1: Descriptive statistics for 'High' and 'Low' clusters of caregiver-rated variables identified in the frequentist analysis

Caregiver-Rated Variable	'High' Cluster Mean (SD)	'Low' Cluster Mean (SD)	Difference in Means (95% Confidence Interval)	Significance
Zarit Burden Interview (ZBI)	31.09 (7.04)	10.83 (5.88)	17.03-23.49	$p = 2.2x10^{-16}$ *** t = 12.54
Caregiver-Rated Impulsiveness (BIS)	71.17 (6.30)	54.92 (6.65)	12.9-19.5	$p = 1.3x10^{-13}$ *** t = 9.85
Caregiver-Rated Empathy (EQ)	48.29 (6.26)	27.03 (8.12)	17.66-24.86	$p = 2.2x10^{-16}$ *** t = 11.81

Significance codes: '***' p<0.001 '**' p<0.01 '*' p<0.05

Supplementary Table 2: Levodopa Equivalent Daily Dose (LEDD) at each time point

Time Point	LEDD Mean (SD)	LEDD Median (Range)
Pre-DBS	1066 (±551.8)	1000 (0 – 3450)
2-weeks post-DBS	416 (±243.5)	400 (0 -1210)
6-weeks post-DBS	388 (±251.1)	375 (0 – 1463)
13-weeks post-DBS	346 (±217.1)	346 (0 – 950)
26-weeks post-DBS	346 (±208)	345 (0 – 1000)

Supplementary Figure 1

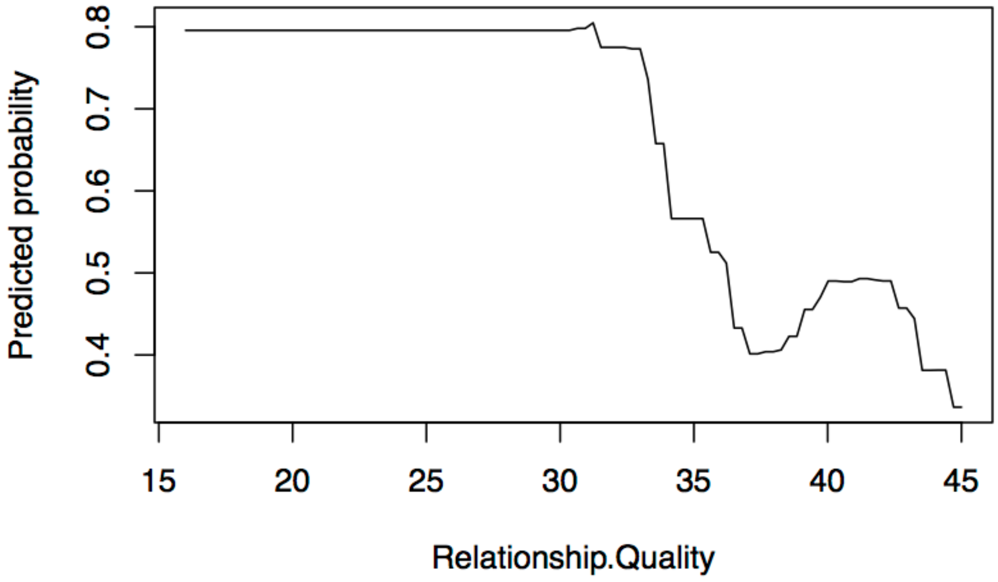

Supplementary Figure 1 Legend:

Likelihood of assignment to the higher burden (ZBI) cluster based on relationship quality (RQI score). Above a certain threshold, assignment to the higher and lower ZBI clusters is of approximately equal likelihood. However, below this threshold, caregivers were far more likely to be assigned to the higher ZBI cluster.
