# Supplementary figures and images for: Caregiver burden and caregiver appraisal of psychiatric symptoms are not modulated by subthalamic deep brain stimulation for Parkinson’s disease
Source: NPJ Parkinsons Dis. 2018 Apr 17;4:12. doi: 10.1038/s41531-018-0048-2 (PMC5904120; doi:10.1038/s41531-018-0048-2)

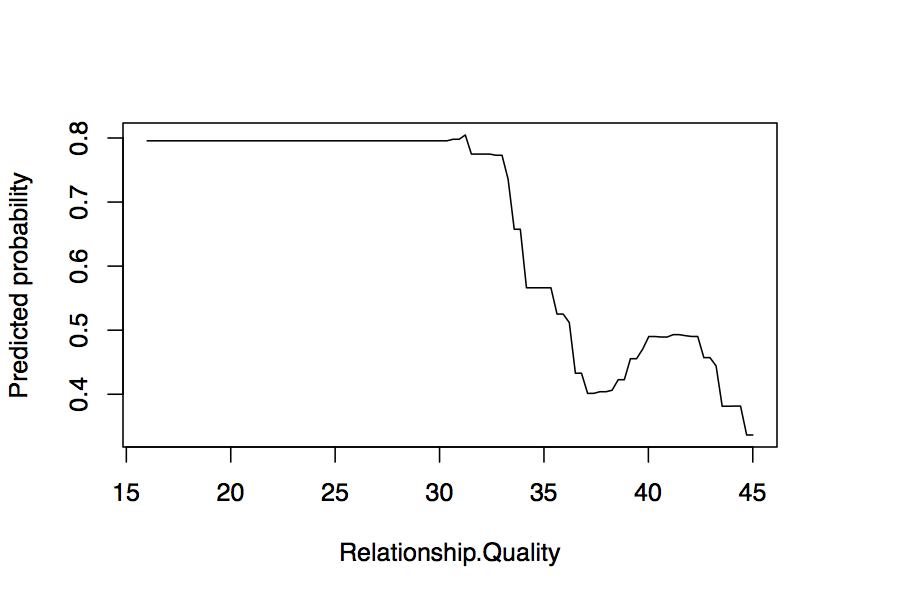

Supplement: Supplementary file 2 — Supplementary Figure 1 [file 41531_2018_48_MOESM2_ESM.tif]
